# Primary Extracranial Meningioma of the Sphenoid Bone Without Dural Attachment Presenting With Multicompartmental Extension: A Case Report

**DOI:** 10.7759/cureus.114473

**Published:** 2026-08-13

**Authors:** Yuki Sakaeyama, Yutaka Fuchinoue, Shuhei Kubota, Mitsuyoshi Abe, Nobuo Sugo

**Affiliations:** 1 Department of Neurosurgery, Faculty of Medicine, Omori Medical Center, Toho University, Tokyo, JPN; 2 Department of Neurosurgery, Faculty of Medicine, Sakura Medical Center, Toho University, Sakura City, JPN

**Keywords:** ectopic meninigoma, meningioma, preoperative radiological diagnosis, primary extracranial meningioma, sphenoid ridge

## Abstract

Primary extracranial meningiomas are rare tumors that arise outside the cranial cavity without continuity with intracranial structures, and their preoperative diagnosis is often challenging. We report a rare case of a primary extracranial meningioma centered on the sphenoid bone with multicompartmental extension.

A 53-year-old woman presented with a gradually enlarging mass in the left temporal region. Imaging revealed a well-defined lesion extending from the sphenoid sinus to the lateral orbit and temporal region. Computed tomography (CT) demonstrated bone thinning and remodeling without destructive changes. Magnetic resonance imaging showed homogeneous contrast enhancement without parenchymal invasion or peritumoral edema. Notably, iodine-123 N-isopropyl-p-iodoamphetamine (¹²³I-IMP) single-photon emission CT (SPECT) showed no significant tracer uptake, and three-dimensional CT angiography revealed no abnormal tumor vascularity.

The tumor was resected via a frontotemporal craniotomy. No continuity with the dura mater was identified intraoperatively. Histopathological examination demonstrated whorl formation and numerous psammoma bodies, and immunohistochemical findings confirmed a World Health Organization grade 1 transitional meningioma with a low Ki-67 labeling index. Tumor infiltration was observed within both trabecular bone and, in a separate specimen, the temporalis muscle, supporting an extracranial origin. This case highlights that primary extracranial meningioma should be considered in the differential diagnosis of skull base lesions showing non-destructive bony remodeling, homogeneous enhancement, absence of tracer uptake on ¹²³I-IMP SPECT, and lack of dural continuity. For accurate diagnosis, integration of imaging, intraoperative findings, and histopathology is essential.

## Introduction

Meningiomas are tumors arising from meningothelial cells of the arachnoid granulations and represent one of the most common non-glial neoplasms of the central nervous system [1]. They typically develop as intracranial tumors attached to the dura mater; however, in rare cases, they occur outside the cranial cavity and are classified as extracranial meningiomas [2].

Extracranial meningiomas account for approximately 1%-2% of all meningiomas. Most are secondary lesions resulting from direct extension of an intracranial tumor, whereas primary extracranial meningiomas are exceedingly rare [2,3]. Primary extracranial meningiomas are defined by the absence of continuity with intracranial structures. Their pathogenesis remains unclear, and several histogenetic mechanisms have been proposed, including ectopic arachnoid cap cell rests displaced along cranial nerve sheaths or entrapped within skull base foramina during embryologic development, as well as differentiation from undifferentiated mesenchymal cells [4].

These tumors most frequently arise in the head and neck region, particularly in the nasal cavity and paranasal sinuses, orbit, temporal bone, scalp, and parapharyngeal region. Histologically and immunohistochemically, they closely resemble intracranial meningiomas, typically demonstrating epithelial membrane antigen (EMA) positivity and similar histological subtypes, making clinicoradiological correlation essential for accurate diagnosis [1,4]. However, preoperative diagnosis is often difficult because clinical symptoms and imaging findings are nonspecific [1,5]. Meningiomas involving the paranasal sinuses, especially the sphenoid sinus, are extremely rare and may mimic inflammatory diseases or other neoplasms, making accurate diagnosis challenging [6].

Herein, we report an extremely rare case of primary extracranial meningioma centered on the left sphenoid bone, extending into the sphenoid sinus, lateral orbit, and temporal region without dural continuity. This case highlights the importance of including this entity in the differential diagnosis of paranasal sinus and skull base tumors.

## Case presentation

A 53-year-old woman presented to the dermatology department of our hospital with a gradually enlarging mass in the left temporal region that she had noticed several years earlier. Magnetic resonance imaging (MRI) revealed a continuous mass lesion extending from the left orbit to the sphenoid sinus, and she was referred to the otolaryngology department. An endoscopic transnasal biopsy of the sphenoid sinus lesion was performed, and histopathological examination confirmed the diagnosis of meningioma. She was subsequently referred to our department for further evaluation and treatment. On admission, her visual acuity was 1.2 bilaterally, and no neurological deficits, including visual field disturbance or diplopia, were observed.

CT findings

**Figure 1:**
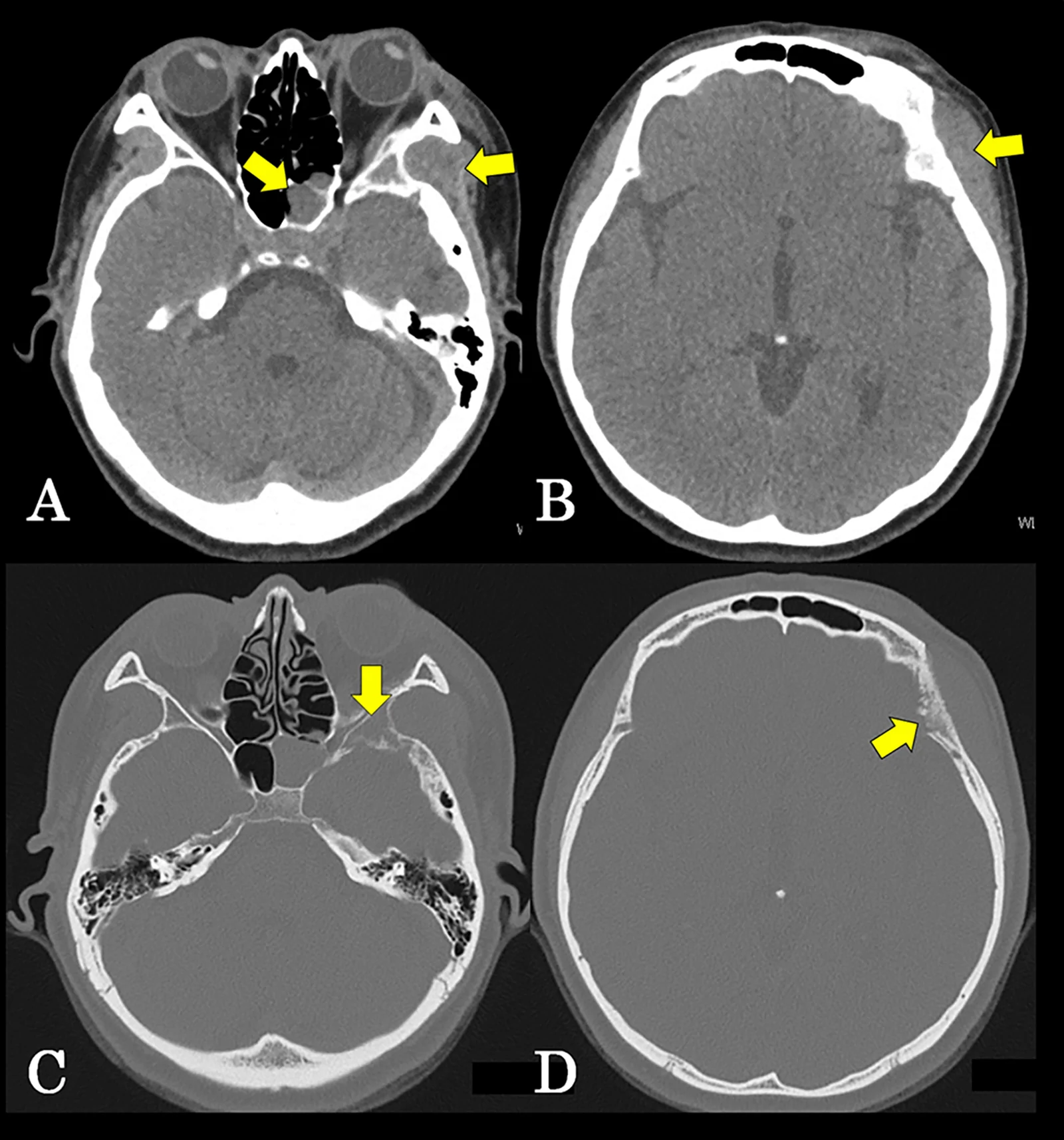
Computed tomography findings Axial CT images at (A) the level of the sphenoid sinus demonstrating a mass lesion occupying the sphenoid sinus and (B) the level of the orbit and temporal region showing tumor extension to the lateral orbit and temporal region. Arrows indicate the tumor. (C, D) Bone window CT images demonstrating mild thinning and remodeling of the left sphenoid bone and part of the left frontal bone without evidence of overt bone destruction. Arrows indicate bone abnormalities.

CT demonstrated a mass lesion extending from the sphenoid sinus to the lateral orbit and temporal region (Figures 1A, 1B). Bone window CT showed mild thinning and remodeling of the left sphenoid bone and a portion of the left frontal bone, without evidence of overt bone destruction (Figures 1C, 1D).

MRI findings

**Figure 2:**
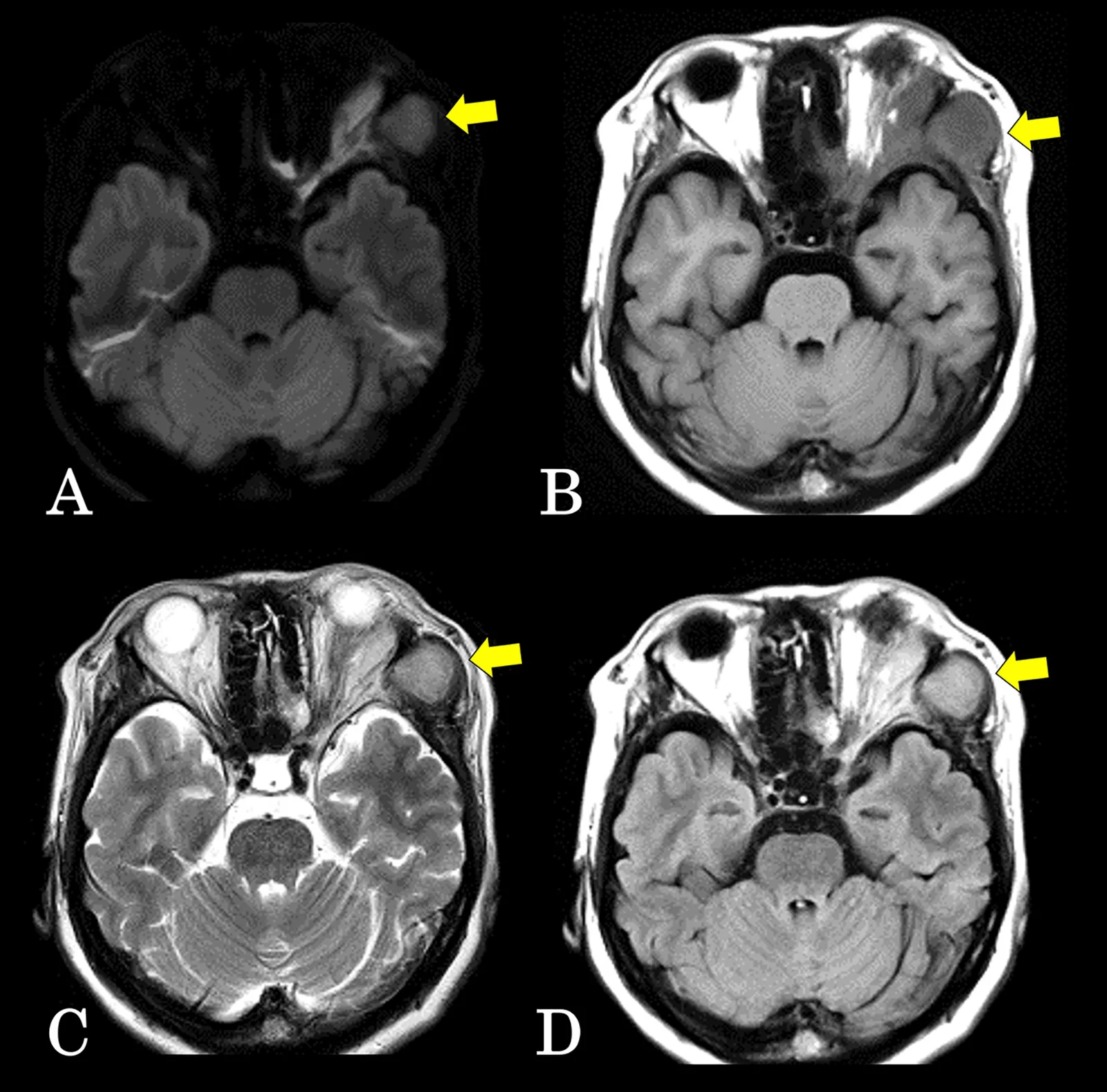
Magnetic resonance imaging findings (non-contrast). Arrows indicate the tumor. (A) Diffusion-weighted imaging showing no apparent diffusion restriction. (B) T1-weighted image demonstrating an iso- to slightly hypointense signal intensity. (C) T2-weighted image showing a slightly hyperintense signal. (D) Fluid-attenuated inversion recovery (FLAIR) image demonstrating a mildly increased signal intensity without surrounding edema.

On MRI, the lesion did not show apparent diffusion restriction on diffusion-weighted imaging (DWI) (Figure 2A). It appeared iso- to slightly hypointense on T1-weighted images (Figure 2B) and slightly hyperintense on T2-weighted and FLAIR images (Figures 2C, 2D). No significant peritumoral edema or parenchymal invasion was observed.

**Figure 3:**
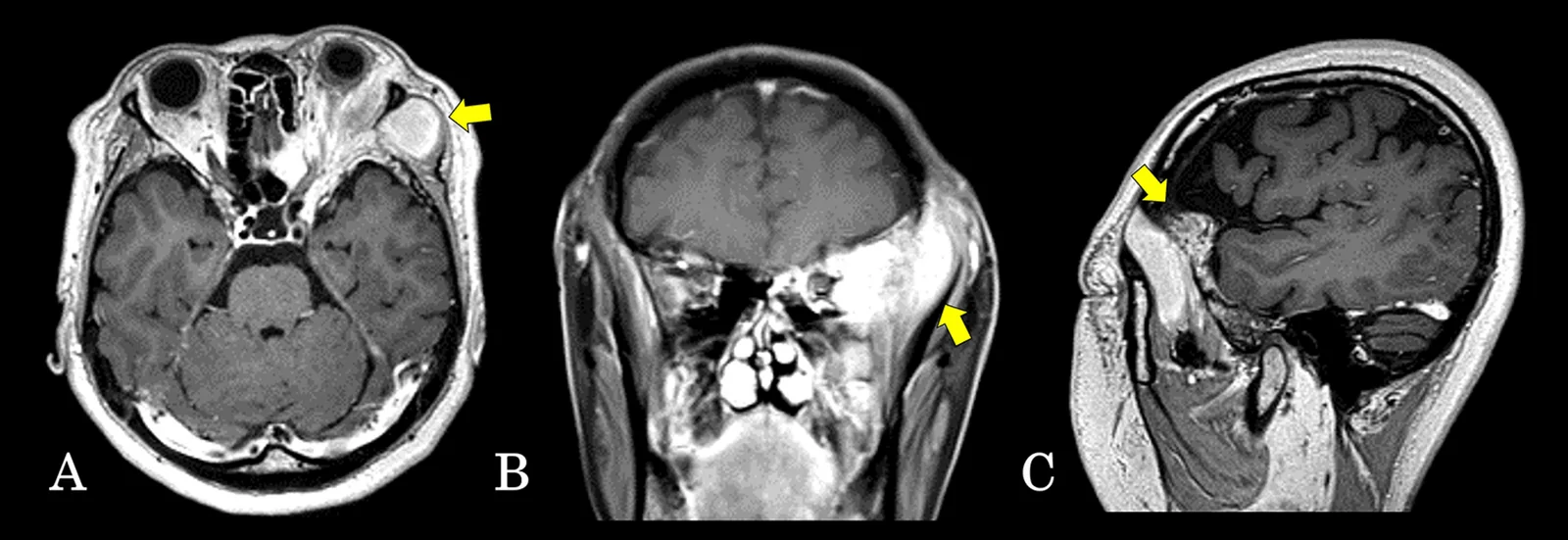
Contrast-enhanced magnetic resonance imaging findings. Arrows indicate the tumor. (A) Axial contrast-enhanced T1-weighted image. (B) Coronal contrast-enhanced T1-weighted image. (C) Sagittal contrast-enhanced T1-weighted image. These images demonstrate a well-defined, homogeneously enhancing mass centered on the left sphenoid bone, extending into the sphenoid sinus, lateral orbit, and temporal region, without evidence of brain parenchymal invasion.

Contrast-enhanced T1-weighted MRI revealed a well-defined, homogeneously enhancing mass centered on the left sphenoid bone, extending into the sphenoid sinus, lateral orbit, and temporal region (Figures 3A-3C). No invasion into the brain parenchyma was identified.

Single-photon emission CT findings

**Figure 4:**
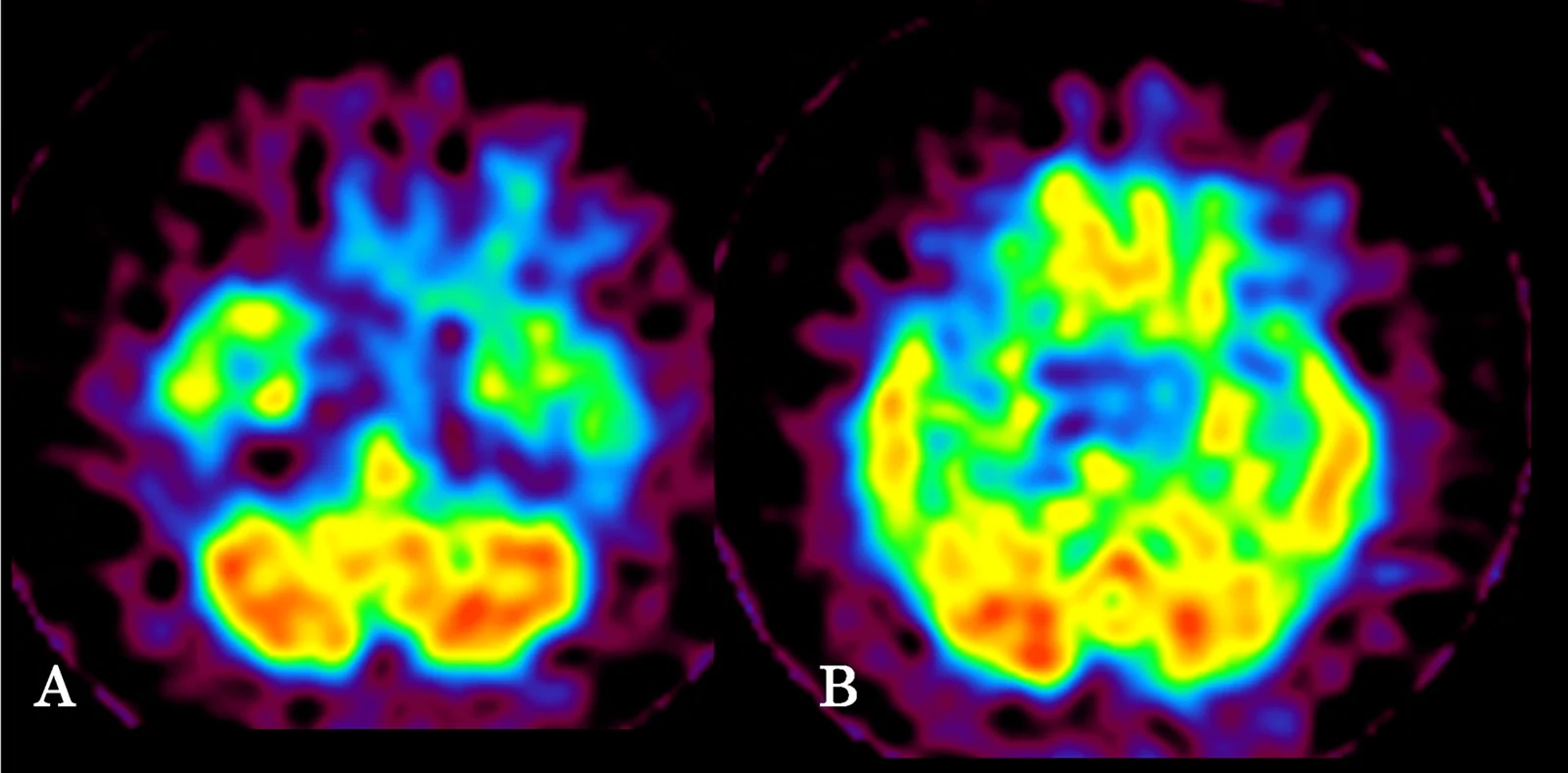
Single-photon emission computed tomography (SPECT) findings SPECT images at (A) the level of the paranasal sinuses and (B) the level of the temporal region. These images show no significant tracer accumulation corresponding to the tumor in the left sphenoid region and no apparent perfusion abnormalities in the surrounding brain parenchyma.

On iodine-123 N-isopropyl-p-iodoamphetamine (¹²³I-IMP) single-photon emission CT (SPECT), no significant tracer accumulation corresponding to the tumor in the left sphenoid region was observed (Figures 4A, 4B). Additionally, no apparent perfusion abnormalities were detected in the surrounding brain parenchyma.

Three-dimensional CT angiography findings

**Figure 5:**
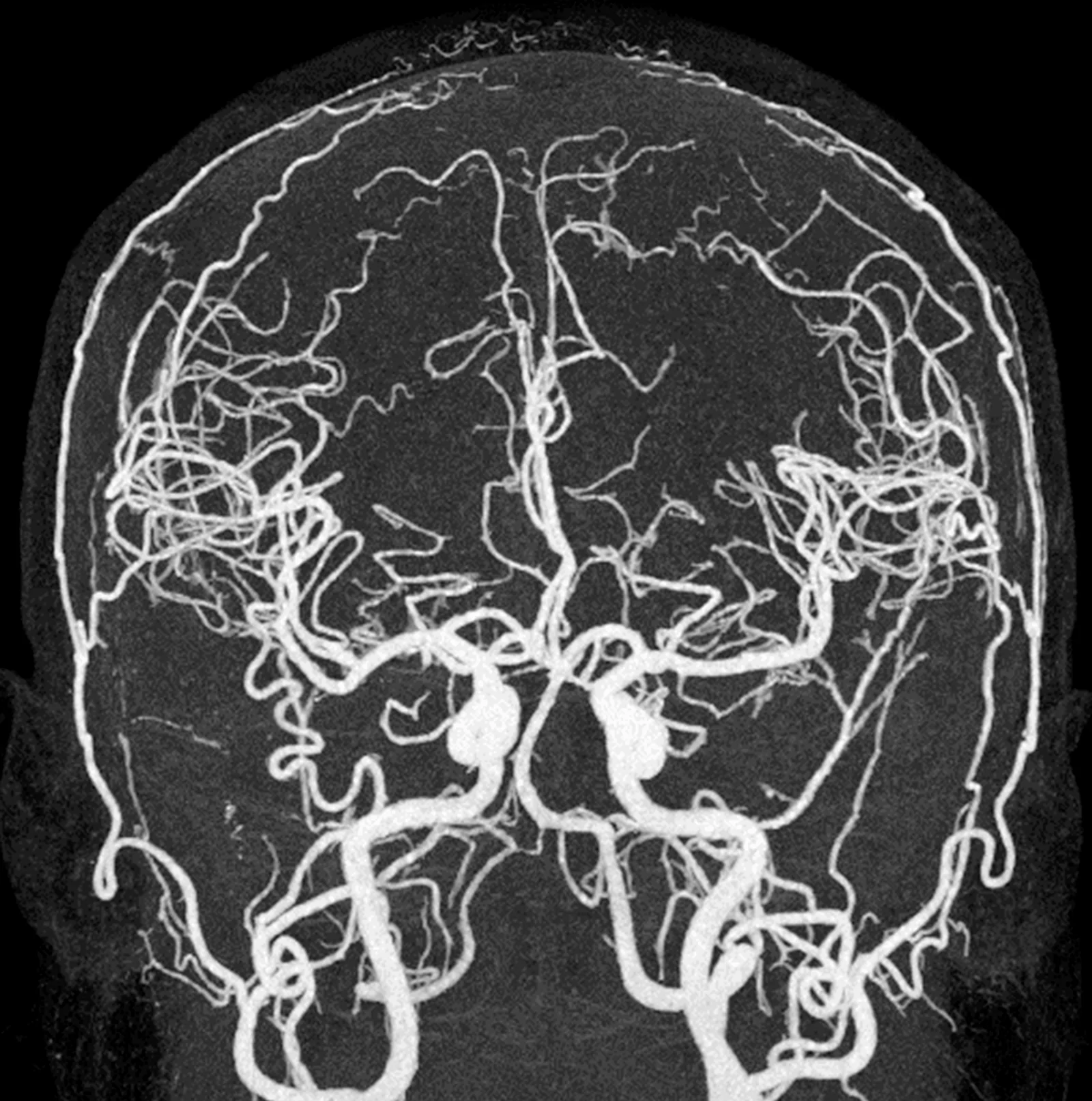
Three-dimensional computed tomography angiography demonstrating no abnormal tumor vascularity or tumor staining and no involvement of major vessels

Three-dimensional CT angiography (3D-CTA) revealed no abnormal tumor vascularity or evident tumor staining corresponding to the lesion (Figure 5).

Surgical procedure

**Figure 6:**
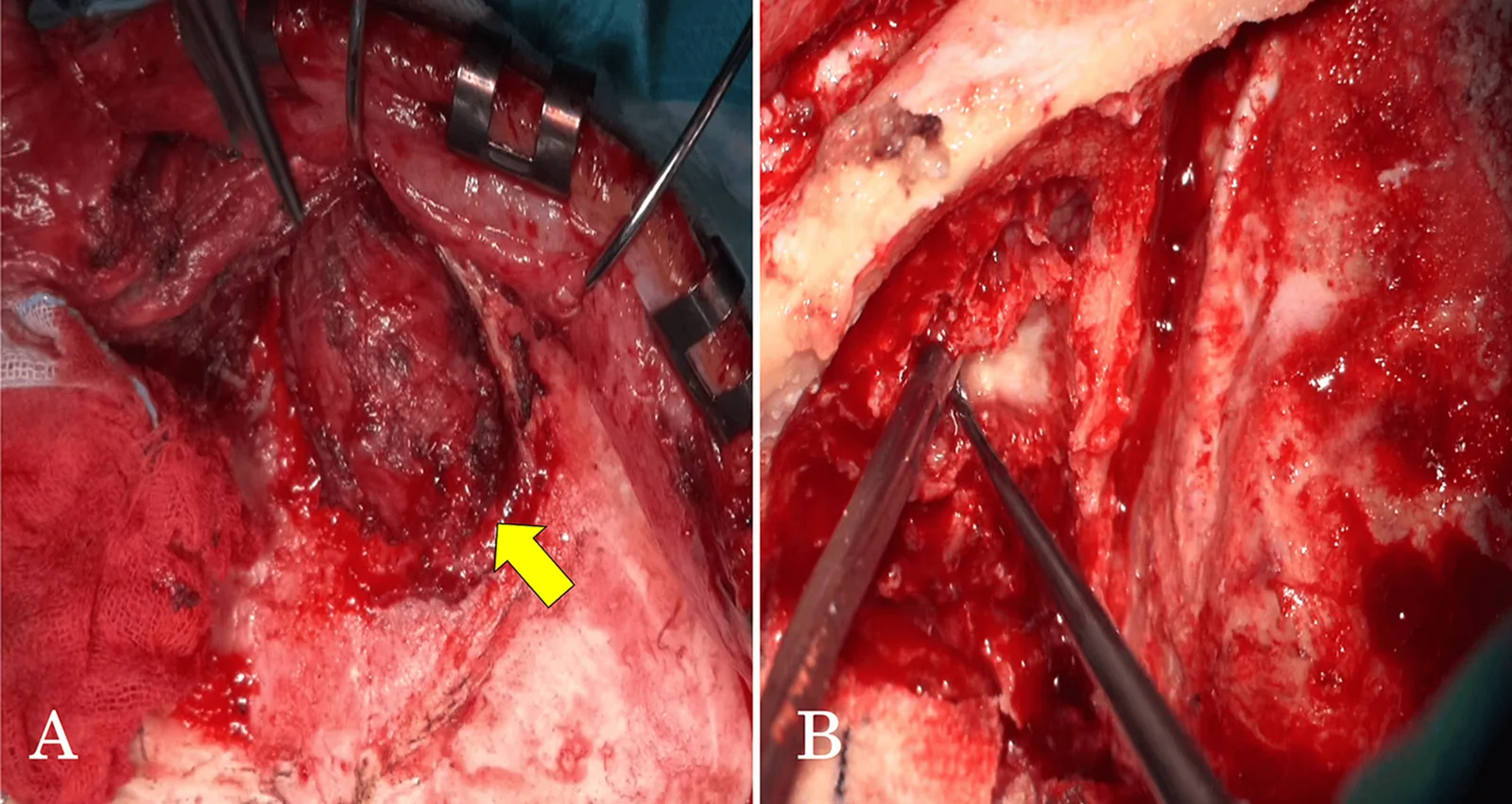
Intraoperative findings (A) Intraoperative view showing the tumor located within the temporalis muscle and extending toward the lateral orbit. The arrow indicates the tumor. (B) Intraoperative view after resection of the tumor in the infratemporal fossa.

Preoperative tumor embolization was not performed because 3D-CTA demonstrated no apparent feeding vessels or tumor staining, and the anticipated benefit of embolization was considered limited. Tumor resection was performed under general anesthesia via a frontotemporal craniotomy approach. The tumor was located within the temporalis muscle and extended to the lateral orbit (Figure 6A). It was carefully dissected and removed while preserving the surrounding structures. Subsequently, anterior clinoidectomy and removal of the surrounding orbital bone were performed to access and resect the tumor involving the sphenoid bone. No obvious invasion into the periorbita was observed. Finally, the tumor located in the infratemporal fossa was resected (Figure 6B). The deeply situated tumor extending into the sphenoid sinus was partially left in place considering surgical safety, and the procedure was concluded. Intraoperatively, no areas of tumor continuity with the dura mater were identified. No cerebrospinal fluid leakage was observed during surgery. Postoperatively, the patient experienced transient ocular motility disturbance, which resolved spontaneously. The swelling in the left temporal region subsided. The residual lesion was evaluated using contrast-enhanced MRI. Follow-up contrast-enhanced MRI performed at six months and one year postoperatively demonstrated no enlargement of the residual tumor within the sphenoid sinus and no obvious recurrence. No neurological deficits were observed during follow-up.

Pathological findings

**Figure 7:**
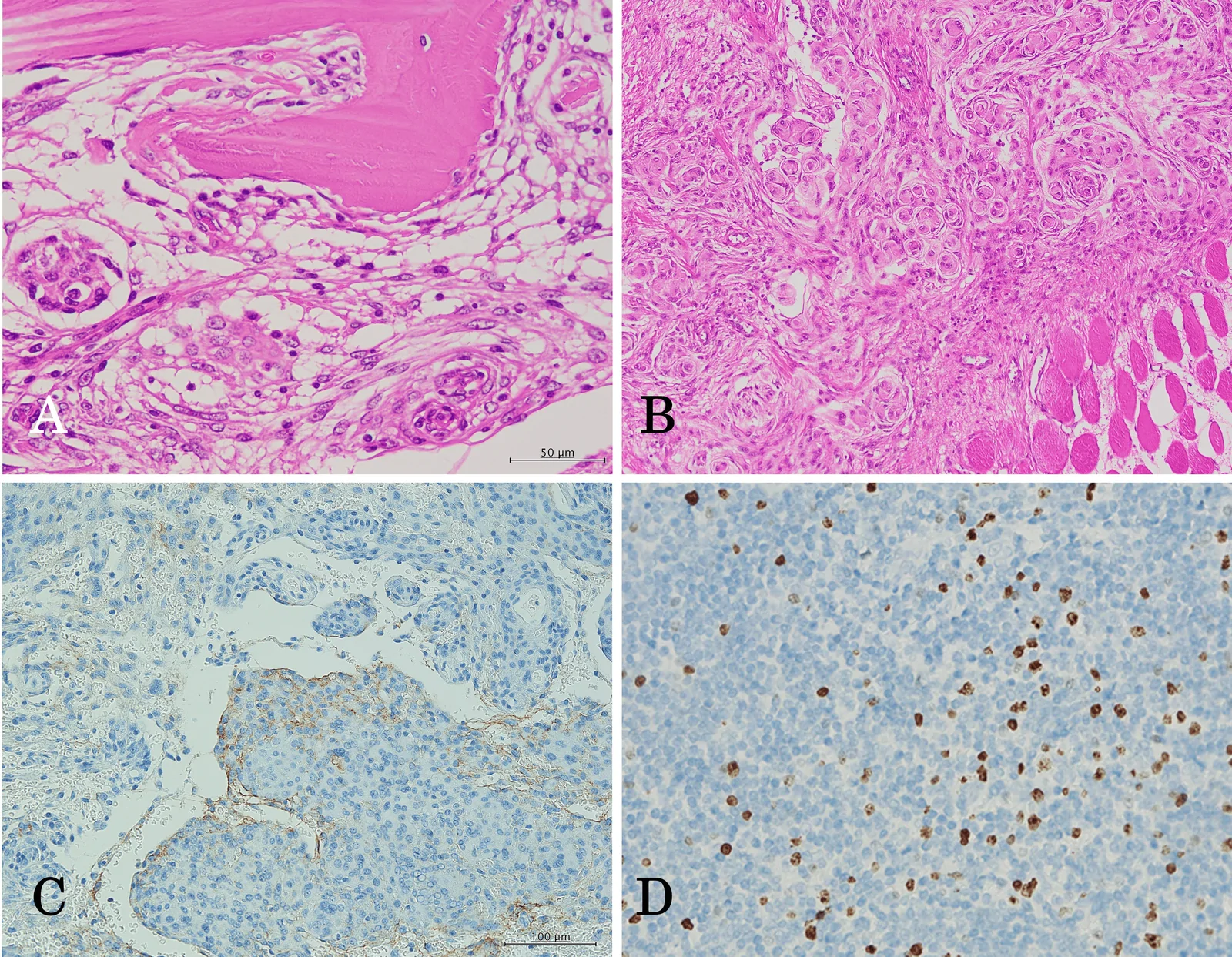
Histopathological findings (A) H&E staining showing tumor cell infiltration within the trabecular bone (×400). (B) H&E staining from a separate specimen showing tumor infiltration within the temporalis muscle (×100). (C) EMA staining showing tumor cell positivity (×100). (D) The Ki-67 index showing a low labeling index (×200).

Hematoxylin and eosin (H&E) staining revealed a tumor composed of round to spindle-shaped cells proliferating within a fibrous stroma. At high magnification (×400), tumor cell infiltration within and around the trabecular bone was observed, suggesting an intraosseous origin (Figure 7A). In contrast, a specimen obtained from a separate site (Figure 7B, magnification ×100) demonstrated tumor infiltration within the temporalis muscle, with tumor cells distributed between skeletal muscle fibers. The tumor cells showed prominent whorl formations and numerous concentrically laminated calcified structures (psammoma bodies). The nuclei were relatively uniform, round to oval, with minimal atypia. No mitotic figures or necrosis were identified (Figure 7B). Immunohistochemically, the tumor cells were positive for EMA, showing membranous and cytoplasmic staining, supporting meningothelial differentiation (Figure 7C). Focal progesterone receptor (PgR) positivity was also observed. The Ki-67 labeling index was low (approximately 1%-3%), indicating low proliferative activity (Figure 7D).

Based on these findings, the tumor was diagnosed as a transitional meningioma with prominent psammoma bodies.

## Discussion

The present case is noteworthy for two reasons. First, the lesion was centered on the sphenoid bone and showed multicompartmental extension to the sphenoid sinus, lateral orbit, temporalis muscle, and infratemporal fossa. Second, neither imaging nor intraoperative inspection demonstrated continuity with the dura mater. These features are important in distinguishing a primary extracranial meningioma from the secondary extracranial extension of an intracranial tumor [2]. Primary extracranial meningiomas have been reported in various anatomical locations, including the orbit, scalp, paranasal sinuses, nasal cavity, temporal bone, and parapharyngeal region. Histologically, they closely resemble intracranial meningiomas, with meningothelial, fibrous, and transitional subtypes being the most common. Typical pathological features include whorl formation, psammoma bodies, and EMA positivity. Complete surgical resection is the treatment of choice and is generally associated with a favorable prognosis, although recurrence may occur following incomplete resection or in tumors with more aggressive pathological features [4]. Although primary extracranial meningiomas involving the sphenoid bone or sphenoid sinus have been reported, they remain extremely rare. Previously reported cases have also been managed primarily by surgical resection, with favorable clinical outcomes in most reported patients. Among these reports, the present case is distinctive because the tumor was centered on the sphenoid bone with simultaneous extension into the sphenoid sinus, lateral orbit, temporalis muscle, and infratemporal fossa, without radiological or intraoperative evidence of dural attachment. Owing to the involvement of critical skull base structures, subtotal resection was selected to preserve function, and no recurrence has been observed during the one-year follow-up [5,6].

In the diagnostic workup, the pattern of bony involvement was informative. CT demonstrated thinning and remodeling of the sphenoid and frontal bones without overt destruction, which differs from the aggressive bone destruction typically seen in malignant tumors [1]. MRI showed homogeneous contrast enhancement without parenchymal invasion or peritumoral edema, findings suggestive of a low-grade lesion [5]. However, these imaging features are not specific, and a definitive diagnosis cannot be established based on imaging alone.

In this case, ¹²³I-IMP SPECT and 3D-CTA provided additional diagnostic information. The absence of tracer accumulation on ¹²³I-IMP SPECT was particularly relevant, as primary central nervous system lymphoma (PCNSL) is known to demonstrate high uptake or retention on this modality [7,8]. Therefore, PCNSL was considered less likely. However, because the absence of ¹²³I-IMP uptake is not specific for primary extracranial meningioma, this finding should be interpreted cautiously and in conjunction with other radiological and intraoperative findings. Furthermore, 3D-CTA revealed no abnormal tumor vascularity or involvement of major vessels, making hypervascular tumors less likely. Taken together, these findings helped narrow the differential diagnosis.

The diagnosis of primary extracranial meningioma was ultimately established through integration of radiological, intraoperative, and pathological findings. Imaging demonstrated a lesion centered on the sphenoid bone, and no continuity with intracranial structures was identified intraoperatively. Because the tumor was centered within the sphenoid bone and extended secondarily into the sphenoid sinus and adjacent extracranial soft tissues without dural attachment, we interpreted this lesion as a primary extracranial meningioma, most likely originating within the sphenoid bone, rather than secondary osseous involvement by an intracranial meningioma. Histopathological examination demonstrated tumor infiltration within trabecular bone and, in a separate specimen, within the temporalis muscle. Previous studies have shown that bone involvement may occur in both primary intraosseous meningiomas and intracranial meningiomas with secondary osseous invasion, and therefore does not by itself establish the site of origin [9]. In the present case, the diagnosis of a primary extracranial meningioma was based on the integration of radiological, intraoperative, and histopathological findings. Specifically, the lesion was centered on the sphenoid bone, no radiological or intraoperative evidence of dural continuity was identified, and tumor infiltration was observed within the trabecular bone. Taken together, these findings favored a primary extracranial meningioma originating within the sphenoid bone rather than secondary osseous involvement by an intracranial meningioma. In addition, the presence of whorl formation, numerous psammoma bodies, EMA positivity, and a low Ki-67 labeling index is consistent with a World Health Organization grade 1 transitional meningioma. 

From a therapeutic perspective, surgical resection remains the mainstay of treatment for extracranial meningiomas [1]. However, in cases involving critical structures such as the skull base and orbit, subtotal resection may be appropriate to preserve function. In the present case, the deeply situated tumor extending into the sphenoid sinus was partially preserved for safety reasons. Given the low proliferative activity of the tumor, this strategy appears reasonable, although long-term follow-up is required due to the potential risk of recurrence after incomplete resection [1]. Bone involvement has also been reported to be associated with an increased risk of recurrence, further supporting the need for careful long-term radiological follow-up. Despite the extensive multicompartmental extension involving the sphenoid sinus, lateral orbit, temporalis muscle, and infratemporal fossa, the patient had no preoperative neurological deficits. This may be attributable to the slow-growing nature of this World Health Organization grade 1 meningioma, which allowed gradual expansion along extracranial tissue planes without significant compression or invasion of adjacent neural structures.

This case highlights that primary extracranial meningioma should be considered in the differential diagnosis of skull base lesions demonstrating non-destructive bony remodeling, homogeneous enhancement, and absence of dural continuity. Integration of imaging, intraoperative findings, and histopathology is essential for accurate diagnosis [1,5]. A limitation of the present case is that 68Ga-DOTATATE PET-CT, which may provide additional diagnostic information in meningioma evaluation, was not performed.

## Conclusions

This case report describes a rare primary extracranial meningioma of the sphenoid bone with multicompartmental extension and no dural continuity. Non-destructive bone remodeling, homogeneous MRI enhancement without invasion, and operative findings facilitated differentiation from other skull base tumors. Although the absence of ¹²³I-IMP uptake may have contributed to the differential diagnosis in the present case, this finding is not specific for primary extracranial meningioma. Diagnosis requires integration of imaging, surgical, and pathological findings. Also, because subtotal resection was performed to preserve function, careful long-term radiological follow-up is warranted.

## References

[1] Rushing EJ, Bouffard JP, McCall S, Olsen C, Mena H, Sandberg GD, Thompson LD (2009). Primary extracranial meningiomas: an analysis of 146 cases. Head Neck Pathol.

[2] Aiyer RG, Prashanth V, Ambani K, Bhat VS, Soni GB (2013). Primary extracranial meningioma of paranasal sinuses. Indian J Otolaryngol Head Neck Surg.

[3] Sigdel K, Ding ZF, Xie HX (2022). Case of primary extracranial meningioma of the maxillary sinus presenting as buccal swelling associated with headache: a case report. World J Clin Cases.

[4] Iaconetta G, Santella A, Friscia M, Abbate V, Califano L (2012). Extracranial primary and secondary meningiomas. Int J Oral Maxillofac Surg.

[5] De Pascale A, Ferruccio M, Giuseppe P, Gabriele A, Palumbo A, Trecca EM (2025). Diagnostic challenges of sinonasal extracranial meningiomas: a case report. Acta Biomed.

[6] Xiong J, Li N, Wu Y (2024). Primary typical meningioma of the sphenoid sinus: a case report and literature review. Indian J Otolaryngol Head Neck Surg.

[7] Akiyama Y, Moritake K, Yamasaki T (2000). The diagnostic value of 123I-IMP SPECT in non-Hodgkin's lymphoma of the central nervous system. J Nucl Med.

[8] Shinoda J, Yano H, Murase S, Yoshimura S, Sakai N, Asano T (2003). High 123I-IMP retention on SPECT image in primary central nervous system lymphoma. J Neurooncol.

[9] Clynch AL, Norrington M, Mustafa MA (2023). Cranial meningioma with bone involvement: surgical strategies and clinical considerations. Acta Neurochir (Wien).

